# Contralateral Renal Cell Carcinoma Ureteric Metastases Can Arise on Tyrosine Kinase Adjuvant Therapy and Be Effectively Treated by Endoscopic Laser Excision and Ablation

**DOI:** 10.1155/2014/359352

**Published:** 2014-07-01

**Authors:** Sarah L. Reid, Nikolas J. Arestis, Craig McIlhenny, Gavin W. A. Lamb

**Affiliations:** Forth Valley Royal Hospital, Stirling Road, Larbert FK5 4WR, UK

## Abstract

Renal cell carcinoma (RCC) uncommonly metastasizes to the ureter and rarely to the contralateral ureter. We describe the presentation of 2 successive contralateral ureteric metastases from RCC in our institution. The first represents the only reported metachronous ureteric deposit on adjuvant sorafenib after laparoscopic radical nephrectomy for RCC. The other presented with a synchronous lesion after radiological work-up. Both lesions were treated with endoscopic excision and laser ablation with preservation of the renal unit and no local recurrence. We report these cases and discuss the literature.

## 1. Introduction

Ureteric metastasis from renal cell carcinoma (RCC) is a rare finding; ipsilateral lesions are the most reported in literature with over 50 reported to date. We describe the presentation and management of two very different cases of contralateral ureteric metastases. The majority of literature describes surgical excision of these lesions. We outline a novel treatment of these rare lesions with endoscopic biopsy and laser ablation in order to preserve renal function and reduce morbidity.

## 2. Case Reports

### 2.1. Case 1

**Figure 1:**
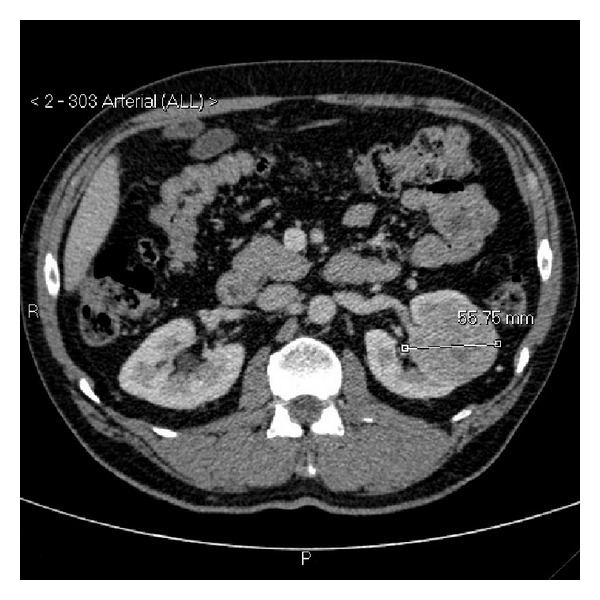
Axial portal venous phase CT scan at initial presentation demonstrating a 56 mm mass of the interpolar left kidney. Normal left renal vein.

54-year-old male presented in 2010 with painless visible haematuria. No significant past medical history was reported other than hypercholesterolaemia. Ultrasound revealed a left renal mass; however, CT scan demonstrated in addition to the 56 mm left renal mass an 8 mm right pelviureteric junction stone in the lower pole moiety of a partial duplex kidney joining in the proximal third of the ureter (Figure 1).

There was no evidence of metastatic disease and accordingly he underwent right flexible ureteroscopy laser lithotripsy of calculus and stent insertion. Four weeks later flexible cystoscopy with removal of stent and laparoscopic radical nephrectomy were performed. Pathology revealed a pT1b Fuhrman grade 3 Leibovich score 4 intermediate risk clear cell carcinoma with complete excision. He was recruited to the SORCE adjuvant trial in view of his intermediate risk (a double blind adjuvant trial of sorafenib versus placebo) [1].

**Figure 2:**
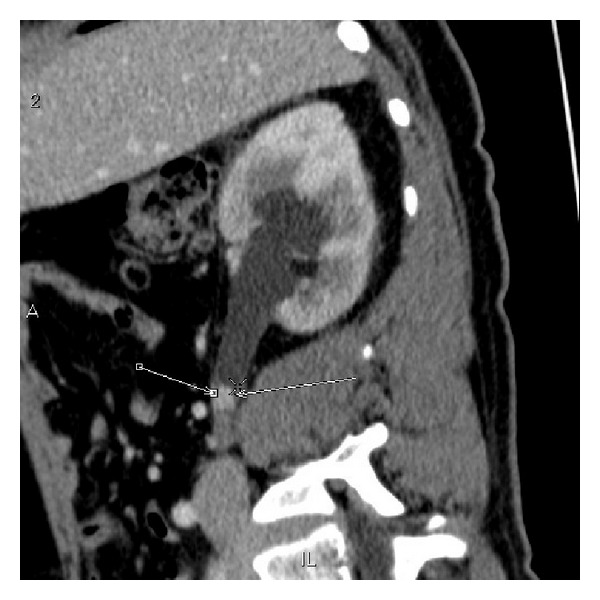
Portal venous phase CT (oblique sagittal MPR) showing an enhancing 9 mm soft tissue lesion (arrows) obstructing the lower moiety ureter right kidney.

He presented 14 months later with visible haematuria and a reduced eGFR of 46. CT-IVU demonstrated a 9 mm enhancing soft tissue lesion obstructing the lower pole moiety of the right kidney partial duplex at a similar position to his previous PUJ calculus (Figure 2). Flexible ureteroscopy demonstrated a smooth surfaced vascular pale pedunculated lesion on a narrow stalk extending into the lumen of the ureter. This was excised and the pedicle base fulgurated using a Holmium laser 200 *μ*m fibre at 10 W and the specimen removed using a 1.9 French ZeroTip Nitinol basket. The ureter was secured with a 6 French 24 cm stent into his lower pole moiety. Recovery was complicated by readmission 7 days after procedure with further haematuria and decline in renal function necessitating temporary nephrostomy into both moieties due to clot obstruction distally. The nephrostomies were removed after a further week and stent was removed at ureteroscopic surveillance 3 months later. Histology confirmed metastatic clear cell cancer. The patient was unblinded from SORCE trial and had been receiving sorafenib at the time of development of his ureteric metastasis which was subsequently discontinued.

The patient went on to develop a solitary metastasis in lung treated with wedge excision 10 months later; however, he remains free of ureteric recurrence 13 months following excision on no systemic therapy.

### 2.2. Case 2

**Figure 3:**
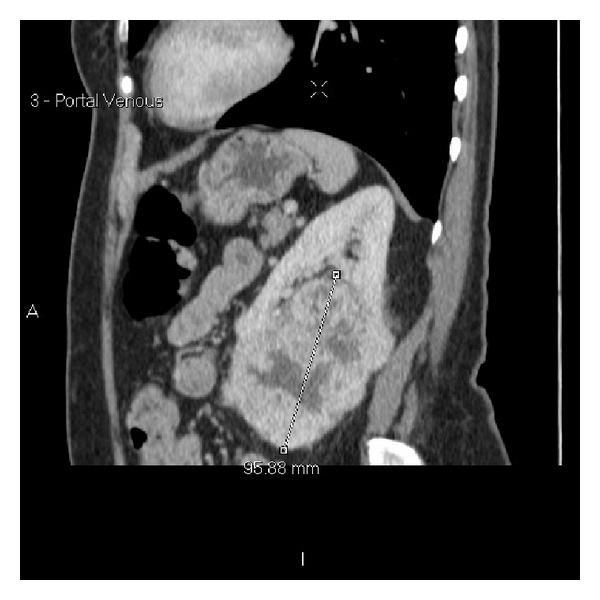
Sagittal portal venous phase staging CT scan demonstrating a 96 mm left renal mass.

**Figure 4:**
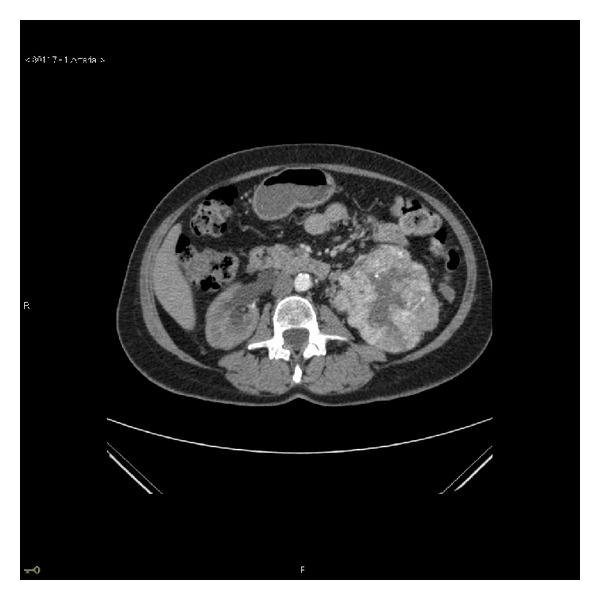
Axial arterial phase staging CT scan again demonstrating a 96 mm left renal mass.

**Figure 5:**
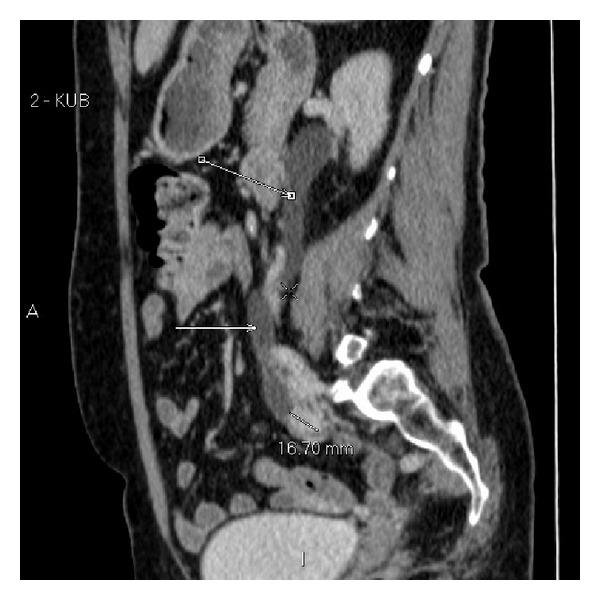
Portal venous phase (split bolus) CT urogram at time of staging. This shows an obstructing, synchronous, enhancing 16 mm soft tissue lesion in the lower third of the right ureter. Arrows demonstrate hydroureter above obstruction.

51-year-old female presented in 2012 with visible haematuria and right sided abdominal pain on a background of recurrent urinary tract infections. Significant past medical history includes hypertension and a subarachnoid haemorrhage in 2006 with full recovery. Ultrasound identified right hydronephrosis in an atrophic kidney and a left renal mass lesion. Staging CT confirmed a 96 mm left renal lesion and a solid obstructing 16 mm intraluminal lesion in the lower third right ureter with no evidence of pulmonary metastases or distant disease (Figures 3, 4, and 5). The size and position of the left renal mass meant that it was not amenable to nephron sparing surgery.

Ureteroscopy confirmed a pedunculated vascular smooth solid lesion identical in appearance to the previous ureteric renal cell metastasis experienced in our unit. Holmium laser excision was performed using the same technique described above. A 6 French 24 cm stent was inserted following excision. Baseline eGFR was 48, DMSA renogram confirmed 9% differential function on the right side, and histology of the lesion revealed metastatic clear cell carcinoma.

The patient was commenced on systemic sunitinib and at 6 months has no evidence of local right ureteric recurrence, no distant disease, and a 10 mm reduction in the primary tumour size. Ureteroscopy has demonstrated no stricturing at the site of metastasectomy to suggest a preexisting condition.

## 3. Discussion

The ureter is an unusual source of metastases with the majority from a nonurological origin. Breast and gastric carcinomas are the commonest nonurologic metastases and bladder and prostate are the commonest urologic metastases [2]. RCC typically metastasizes to lymph nodes, lung, liver, bone, adrenal, and brain but ureteric metastases are reported in over 50 cases. Gelister et al. describe the typical presentation of these lesions after nephrectomy as recurrent haematuria [3]. This is consistent with the cases described above. The postulated mechanisms of spread include intraluminal reflux, haematogenous, or lymphatic spread and are discussed in literature [3–5]. Renal vein involvement increased the chance of ureteric spread from 1.2% to 6.1% in a postmortem series by Saitoh [6].

The influence of retrograde ureteroscopy with stent insertion or local inflammatory response related to endoscopic stone removal in the first case described above is unclear however because of the location of subsequent metastasis is likely to be relevant. The authors hypothesize that this could be either through reflux or through haematogenous spread as described in the literature [3–5]. Patient 2 had an atrophic kidney of unknown aetiology. No radiographic features were present to suggest reflux, nor was there stricturing at the site of ureteric metastasectomy to suggest pre-existing stricture. Despite relief of obstruction, serial renography at 3 and 6 months shows minimal renal recovery of 14% differential function.

It is well accepted that complete metastasectomy of RCC improves patient survival [7]. The majority of cases in the literature involve treatment by surgical excision. In the case of contralateral ureteric metastases, complete excision of the renal unit would make the patient dialysis dependent along with the associated morbidity and mortality. Esrig et al. describe ileal ureteral replacement following total ureterectomy for the management of contralateral ureteric RCC [5]. Our patients underwent endoscopic treatment to preserve renal function. Neither patient has required long term ureteric stenting or developed localised strictures. This has been increasingly described in upper tract transitional cell carcinoma but is novel in the case of ureteric RCC.

In conclusion contralateral ureteral metastases from RCC are a rare occurrence. We describe how novel endoscopic treatment can preserve renal function and reduce morbidity in these complicated patients with metastatic disease. In addition we describe a case where contralateral renal cell ureteric metastasis occurred whilst on adjuvant tyrosine kinase therapy.
